# A randomized clinical trial to assess the influence of a three months training program (Gym-based individualized vs. Calisthenics-based non-invidualized) in COPD-patients

**DOI:** 10.1186/1465-9921-15-36

**Published:** 2014-03-25

**Authors:** Timm Greulich, Katharina Kehr, Christoph Nell, Janine Koepke, Daniel Haid, Ulrich Koehler, Kay Koehler, Silke Filipovic, Klaus Kenn, Claus Vogelmeier, Andreas-Rembert Koczulla

**Affiliations:** 1 Department of Medicine, Pulmonary and Critical Care Medicine, University Medical Center Giessen and Marburg, Philipps-University Marburg, Member of the German Center for Lung Research (DZL), Marburg, Germany; 2 Department for Physiotherapy, University Medical Center Giessen and Marburg, Marburg, Germany; 3 Department of Pulmonology Schön Kliniken, University Medical Center Giessen and Marburg, Berchtesgaden, Germany

## Abstract

**Introduction:**

Pulmonary rehabilitation has been demonstrated to improve exercise capacity, dyspnoea, quality of life and to reduce the adverse effects of acute exacerbations. Current guidelines recommend exercise training in patients with mild to very severe disease. However, there is insufficient data comparing the efficacy of different training approaches and intensities.

**Methods:**

Between January 2009 and December 2012, 105 COPD patients were screened to participate in the study. 61 patients were randomly assigned into an individualized training group or into a non-individualized training group. Both groups exercised once a week for 60 minutes over a time period of three months. At the beginning and after three months, the following measurements were performed: 6-minute walking test (6-MWT), health-related quality of life (St. Georges Respiratory Questionnaire; SGRQ and COPD-Assessment-Test; CAT), M. rectus femoris cross-sectional area, and inflammatory markers in peripheral blood.

**Results:**

Only in the individualized training group we observed a significant change of the 6-MWT (increase of 32.47 m; p = 0.012) and the cross-sectional area of the M. rectus fermoris (increase of 0.57 cm^2^; p = 0.049), while no significant changes occurred in the non-individualized training group. Peroxisome-proliferator-activated receptor-γ coactivator 1α increased in the individualized training only after the three months training period (increase of 0.43 relative copies; p = 0.017), all other myokines and inflammatory markers were not influenced by either of the programs. The total drop-out-rate was 44.3%.

**Conclusion:**

A low frequency outpatient training program may induce modest improvements in exercise capacity and muscle mass only if it is performed on an individualized basis.

## Introduction

Chronic obstructive pulmonary disease (COPD) is characterized by a usually progressive airflow limitation [1]. Extrapulmonary comorbid conditions, like cachexia and muscle atrophy, are frequently observed [2]. Pulmonary rehabilitation has been demonstrated to improve exercise capacity, muscle metabolism, and quality of life [3,4].

Detailed instructions on pulmonary rehabilitation in COPD have been published [5-7]. However, only a limited number of studies compared the impact of various forms and intensities of outpatient training programs in a randomized fashion [8,9].

The role of exercise effects on the peripheral muscle and its myokines has been acknowledged [10]. The underlying mechanism of exercise training includes the expression of the transcription factor peroxisome-proliferator-activated receptor-γ coactivator 1α (PGC1-α) [11-16]. PGC1-α stimulates the expression of FNDC5, a membrane protein that is cleaved and secreted as a newly identified hormone called irisin [17]. The upregulation of PGC1-α has been shown to attenuate inflammation [18].

The most effective frequency of pulmonary rehabilitation is not known [6]. Most pulmonary rehabilitation studies demonstrating benefits are based on at least two sessions per week [19]. However, a recent review article pointed out that only a small proportion of enrolled patients demonstrated continued commitment raising the question whether a once-weekly training program would be associated with better compliance [20].

We conducted a randomized controlled trial to compare the effects of two different training approaches on exercise capacity, QoL, muscle mass, myokines and serum inflammatory markers. Furthermore, screening failure rates and drop-out rates were assessed.

## Methods

### Study outline

**Figure 1:**
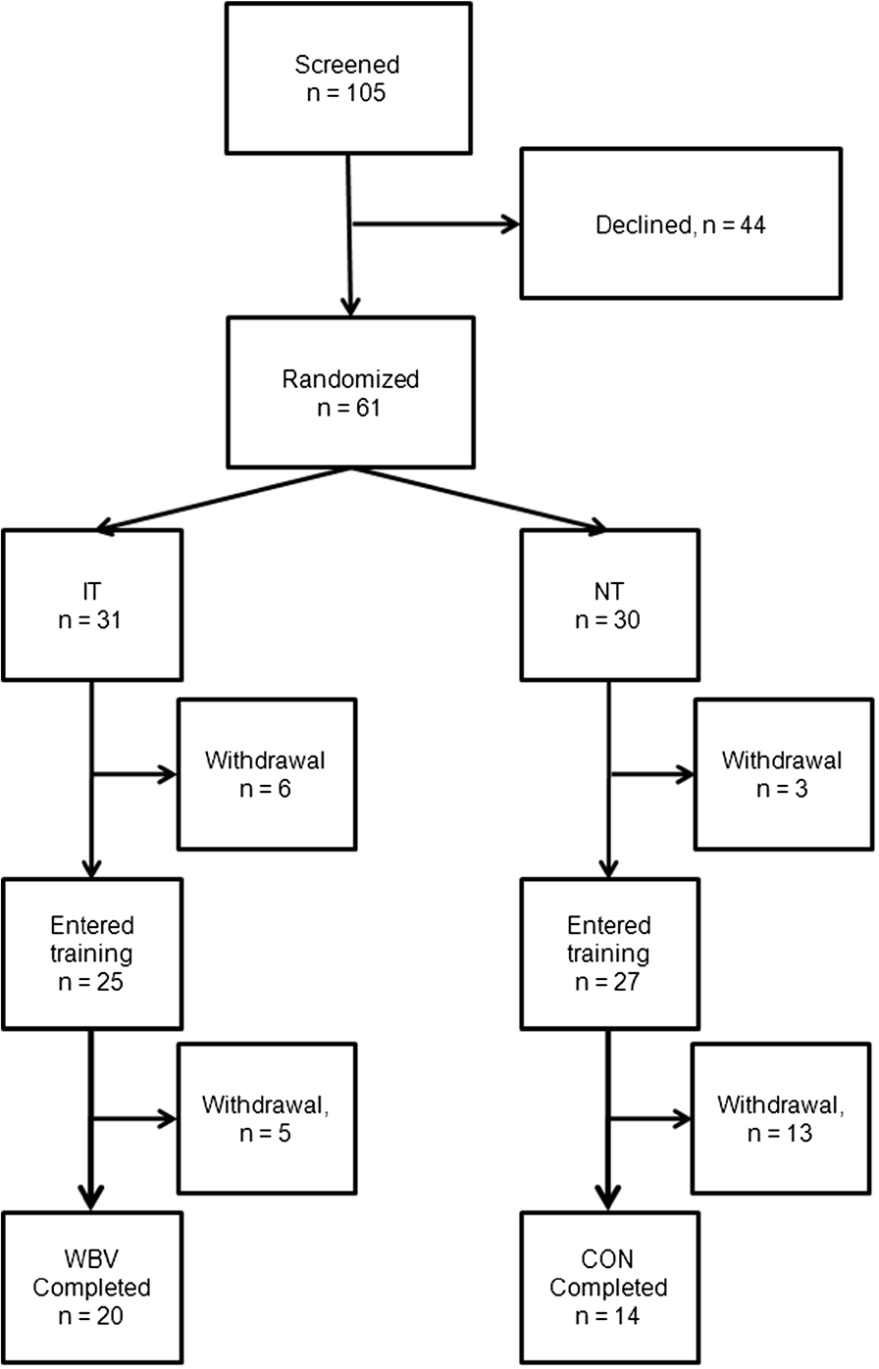
**Trial profile.** 105 stable COPD patients were screened, and 61 patients were randomized in a 1:1 ratio. 34 patients (IT = 20; NT = 14) finalized the program. Abbreviations are explained in the text.

Between January 2009 and December 2012 we invited 105 patients with mild to very severe COPD to participate in our study. We included stable COPD patients based on the diagnostic criteria published by GOLD [1]. Only patients in stable state were considered for randomization. 61 patients agreed to take part in a 3 months ambulatory training program. Patients were randomized to one of the following groups (Trial Flow Figure 1):

– “Individualized Training” (IT): patients participated in a weekly individualized gym-based outpatient exercise training. This training program included all components of exercise training that have been suggested by the joint American College of Chest Physicians and the American Association of Cardiovascular and Pulmonary Rehabilitation (ACCP/AACVPR) clinical practice guidelines [5]. Each patient received an individual training schedule at the beginning of the training period based on his maximal force and endurance time in different approaches. Details can be found in the Additional file 1.

– “Non-individualized Training” (NT): patients participated once weekly as part of a group in different forms of exercise (calisthenics). The training unit was divided into three parts: warm-up (free movements, stretching) for ten minutes; the main part was a forty minute training which included collectively performed exercises like ball games, stepping, thera-band training and dumbbells. The training period was finalized by a ten minute relaxing exercises period. Details can be found in the Additional file 1.

The study was approved by the local ethics committee (Marburg Ethics Committee AZ 123/09, Marburg, Germany).

### Randomization

The randomization was performed by a third party (a statistician from the sleep laboratory of the University of Marburg). A computer generated list was used to produce envelopes that were stored in a locked room. The investigator who wanted to include a patient called the statistician, reported the patient’s identification number and received the allocation to one of both treatment groups.

### Assessments

The following assessments have been performed twice; one time before (M_1_) and one time after the three months training program (M_2_): 6-minute walking test (6-MWT) [21], health-related quality of life using the St. Georges Respiratory Questionnaire (SGRQ) and the COPD-Assessment-Test (CAT), ultrasound measurement of M. rectus femoris cross-sectional area (M. rect. fem.) [22], and serum level measurements of myokines and inflammatory markers.

### Ultrasound

Cross-sectional area of the M. rectus femoris was measured by B-mode ultrasonography as described by Seymour et al. [22]. While patients were in a supine position, the transducer was placed perpendicular to the long axis of the thigh on its superior aspect, three-fifths of the distance from the anterior superior iliac spine to the superior patellar border. The area was calculated as an average of three consecutive measurements.

### Laboratory analyses

White blood cells (WBC), serum levels of C-reactive protein (CRP), interleukin-6 (IL-6), interleukin-8 (IL-8), and tumor necrosis factor-α (TNF-α) were determined. For the quantitative determination of serum irisin concentrations a commercial ELISA kit (Aviscera Bioscience, INC) was used. Serum samples were diluted 1:8 with dilution buffer and measured as duplicate in a plate reader (Tecan infinite® F200pro). The transcription factor PCG1α was analysed in serum using Western Blot. Further details regarding the laboratory analyses can be found in the Additional file 1.

### Statistic and data analysis

The power calculation was done according to the results of Barakat et al. who evaluated an outpatient training program in COPD [23]. We considered our individualized programm to be roughly equally effective (difference in 6-MWT of 46 m). In the above mentioned paper the control group received no intervention and had an increase of 8 m. We anticipated our non-individualized training to be less effective than the individualized but better than a pure control group. We anticipated an increase of the 6-MWT in this group of 27 m (the mean of +46 and +8). We conservatively estimated the standard deviation to be 18 m (twice as high as reported). A power calculation was performed (alpha 0.05, power 0.9) that yielded a group size of 15 patients each (MedCalc 11.1.1.0). Because we expected a drop-out rate of up to 50% we aimed to include 60 patients in the study. All further analysis was calculated with SPSS 21 (IBM Ehningen, Germany) and Prism 5.03 (GraphPad Software, Inc., La Jolla, USA). For comparing two groups the Mann–Whitney-U-Test for unpaired samples was performed, for within-group comparisons a Wilcoxon rank-sum test for paired samples was used. For the investigation of three groups of the Kruskal-Wallis and in the case of ordinally scaled variables, the Fisher exact or chi-square test was used. P-values <0.05 were considered to be significant.

## Results

### Baseline characteristics

61 of 105 invited patients agreed to take part (recruitment rate: 58.1%). The analysis of the baseline characteristics revealed no statistical significant difference between the groups (p > 0.05) at baseline (Table 1). Patients in all groups reported a high number of comorbidities (Table 2).

**Table 1:** Baseline characteristics of all randomized patients

	**IT**	**NT**	**p-value**
**Number**	31	30	N.A.
**Age [years]**	64.61 ± 9.02	65.83 ± 8.59	0.484
**♀/♂**	8/23	14/16	0.113
**BMI [kg/m**^**2**^**]**	26.59 ± 6.61	28.41 ± 6.15	0.920
**FEV**_**1 **_**[% predicted]**	62.01 ± 20.15	68.17 ± 19.8	0.180
**COPD stage [I/II/III/IV]**	6/15/8/2	8/14/7/1	0.876

Continuous variables were tested using Mann–Whitney-U-Test, categorial variables were tested using the Fisher-Test. No significant differences were detected between both groups. Abbreviations are explained in the text.

**Table 2:** Comorbidities (Chart-based)

**Disease**	**IT**	**NT**	**DO**	**Total**
Arterial hypertension	5	7	8	20
Obesity	6	5	9	20
Cardiac diseases	5	2	4	11
Cancer	5	1	2	8
Sleep apnea	3	2	3	8
Diabetes mellitus type 2	1	2	2	5
Hyperlipoproteinemia	2	1	4	7
Depression	2	0	2	4

No statistical significant differences could be detected using Kruskal-Wallis test. Abbreviations are explained in the text.

### Drop-out rate

9 patients (NT: n = 3; IT: n = 6) were dissatisfied with the randomization and did not enter the training. Further 18 patients dropped-out after they attended at least one training session (drop-out rate: 44.3% of all randomized patients). 34 patients completed the study with a final measurement (IT: n = 20; NT: n = 14). There was a differential drop-out rate between the 2 groups if considering only those that entered the training period. In the NT group 51.8% finished the study, whereas in the IT group (80%) ended the trial (p = 0.04). Reasons for discontinuation are displayed in Table 3.

**Table 3:** Drop-out reasons of participants, who attended the training at least one time

**Reasons for quitting program**	**IT**	**NT**
Cardiac disease	0	1
Dissatisfaction with the training program	0	2
Not enough time	1	2
Orthopedic disease	1	1
Participation in an inpatient rehabilitation	0	1
Pneumological disease	0	1
Psychiatric disorder	1	1
Unknown	2	4

No statistical significant differences could be detected using the Fisher-test. Abbreviations are explained in the text.

In a next step we compared the baseline characteristics (Table 4) and baseline measurements (Table 5) of the IT, NT and the drop-out-group (DO). Here, we found differences in lung function, QoL, and serum markers, indicating worse baseline conditions in dropouts.

**Table 4:** Baseline characteristics of dropouts compared to other groups

	**DO**	**IT**	**p-value**^**#**^	**NT**	**p-value**^**##**^	**p-value**^**###**^
**Number**	27	20	N.A.	14	N.A.	N.A.
**Age**	64.15 ± 8.96	65.45 ± 9.35	0.763	66.93 ± 7.76	0.259	0.619
**♀/♂**	13/14	5/15	0.137	4/10	0.321	0.232
**BMI**	28.6 ± 8.44	28 ± 4.62	0.880	29.03 ± 3.29	0.394	0.702
**FEV**_**1**_	57.91 ± 20.93	66.3 ± 20.42	0.333	73.84 ± 16.89	0.039	0.106
**COPD stage I/II/III/IV**	5/11/9/2	5/10/4/1	0.732	4/8/2/0	0.356	0.722

We did not find significant differences between DO vs. IT (^#^), Dropout vs. NT (^##^) or any of the three groups (^###^). Continuous variables were analyzed using Kruskal-Wallis, ordinal-scaled variables were analyzed using Fisher’s exact and Chi square test. Abbreviations are explained in the text.

**Table 5:** Baseline measurements of Dropouts compared to other groups

	**DO**	**IT**	**p-value**^**#**^	**NT**	**p-value**^**##**^	**p-value**^**###**^
**6 MWT [m]**	384.42 ± 98.28	407 ± 105.44	0.345	411.79 ± 64.74	0.318	0.153
**M. rectus femoris [cm**^**2**^**]**	7.07 ± 2.47	6.66 ± 2.7	0.754	7 ± 3.17	1.000	0.903
**SGRQ**	56.19 ± 19.45	46.93 ± 20.72	0.128	37.45 ± 16.92	0.011	0.028
**CAT**	20.04 ± 8.08	19.16 ± 6.37	0.695	14.46 ± 7.37	0.056	0.099
**CRP [mg/dl]**	11.65 ± 14.65	5.61 ± 2.12	0.048	5 ± 0	0.029	0.006
**AAT [g/]**	1.62 ± 0.29	1.38 ± 0.17	0.002	1.44 ± 0.23	0.024	0.005

Analysis included comparison of DO vs. Individualized training (IT, ^#^), Dropout vs. non-individualized training (NT, ^##^) or any of the three groups (^###^). Continuous variables were analyzed using Kruskal-Wallis.

### 6-MWT

**Figure 2:**
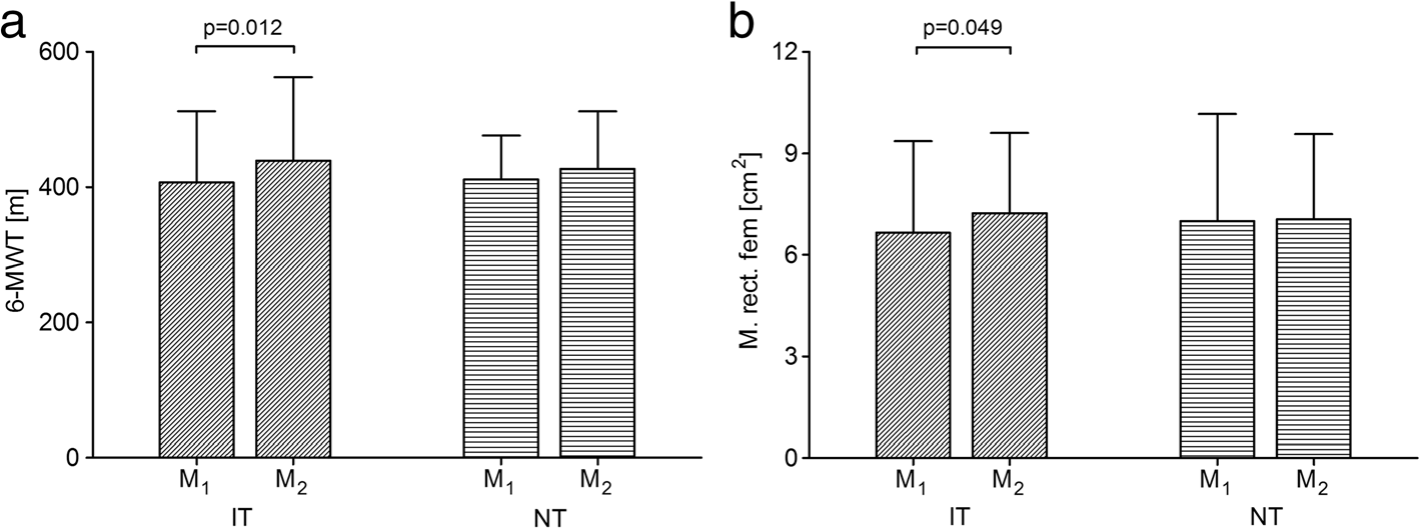
**Exercise capacity and muscle cross-sectional area.** Wilcoxon rank-sum test for paired samples was used to test for significant differences between measurement 1 (M1: before the program) and measurement 2 (M2: after the program).

Analyzing the exercise capacity by using the 6-MWT we saw a significant change of the walking distance in the IT-group (M_1_ = 407 ± 105.44 m, M_2_ = 439.37 ± 122.89 m; p = 0.012, Figure 2a). In the NT group no significant change was observed (M_1_ = 411.79 ± 64.74 m, M_2_ = 427.5 ± 84.57 m; p = 0.116, Figure 2a). No significant between group differences could be observed.

### Ultrasound-measurement of the M. rectus femoris

In the IT-group a significant chance of the cross-sectional area of the M. rectus femoris could be identified (M_1_ = 6.66 ± 2.7 cm^2^, M_2_ = 7.23 ± 2.38 cm^2^; p = 0.049; Figure 2b), whereas this was not the case in the NT group (M_1_ = 7 ± 3.17 cm^2^, M_2_ = 7.05 ± 2.52 cm^2^, p = 0.814, Figure 2b). No significant between group differences could be observed.

### Quality of life

**Figure 3:**
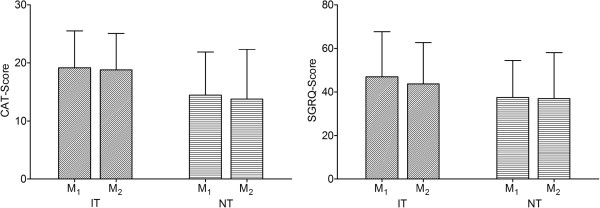
**Quality of life.** Wilcoxon rank-sum test for paired samples was used to test for significant differences between measurement 1 (M1: before the program) and measurement 2 (M2: after the program).

We were not able to detect significant differences of SGRQ and CAT between measurement 1 and 2 in any of the groups (Figure 3).

### Serum markers of inflammation and muscle derived markers

We found no significant differences comparing inflammatory and muscle derived markers between both groups. The values are displayed in Table 6.

**Table 6:** Serum markers of inflammation and myokines

	**IT**		**p-value**	**NT**		**p-value**	**p-value**
	**M**_**1**_	**M**_**2**_		**M**_**1**_	**M**_**2**_		
**CRP [mg/l]**	5.6 + 2.1	6.2 + 2.4	1.000	5 + 0	5.2 + 0.6	0.180	0.419
**WBC [G/l]**	7.5 + 2.1	7.4 + 2.3	0.619	7.2 + 2.1	6.9 + 2.1	0.198	0.666
**IL-6 [ng/l]**	4.9 + 7.9	3.6 + 2.1	0.324	3.8 + 3.1	5.8 + 6.4	0.170	0.653
**IL-8 [ng/l]**	8.4 + 3.0	8.8 + 2.9	0.569	8.8 + 2.8	9.4 + 3.6	0.362	0.691
**TNF-α [ng/l]**	9.9 + 3.5	9.1 + 2.9	0.528	10.7 + 2.9	10.7 + 3.1	0.889	0.587
**PGC1- α [relative copies]**	0.77 ± 0.77	1.2 ± 1.15	0.017	1.03 ± 0.77	1.16 ± 0.81	0.388	0.832
**Irisin [ng/μl]**	128.65 ± 14.87	131.5 ± 33.26	0.528	123.67 ± 17.85	128.86 ± 16.68	0.136	0.138

Wilcoxon rank-sum test for paired samples was used to test for significant differences between measurement 1 (M1: before the program) and measurement 2 (M2: after the program). To compare the deltas (M2-M2) between both groups, the Mann–Whitney-U-Test for unpaired samples was performed. Abbreviations are explained in the text.

## Discussion

To our knowledge this is the first randomized trial comparing a low frequency individualized (IT) vs. non-individualized exercise training (NT) in stable outpatient COPD patients. We found that only IT significantly improved 6-MWT and muscle rectus femoris cross sectional area in a group of patients that exercised once weekly.

Baumann et al. randomized 100 patients with moderate to severe COPD to a continuous outpatient interdisciplinary rehabilitation program or standard care [24]. After 26 weeks, the intergroup difference of the 6-MWT was 59 m in favour of rehabilitation. While the individualized training intervention was similar, the higher intergroup difference compared to our study (32 m) is explained by the different comparator (standard care and non-individualized group training, respectively).

Behnke et al. were able to demonstrate a significant effect of a supervised walking training at home in preserving the hospital-achieved improvement in six-minute walk test and quality of life in patients with severe COPD. 30 out of 46 completed the program and walked 2308 m on 157 days. Thus, the effect was seen in a group of highly compliant patients [25]. No randomization was done in this study. Significant effects have also been observed by du Moulin et al. in patients with moderate COPD. In this randomized trial, ten patients performed home-based exercise training and 10 patients served as controls. After six months the training group had better results than the control group in exercise capacity and lung function [26].

Göhl et al. randomized 34 patients to participate in a multimodular 12 months training program [27]. The training group demonstrated increases in a variety of parameters including the 6-MWT (79 m) and SGRQ (>4 units) whereas in the control group no significant changes were observed. In contrast to our NT group, the intervention included modules of increasing intensity and time, resulting in an increase of 2.4 to 4.2 hours of training per week [27]. The higher intensity and the longer period of time may very well explain larger effects.

The cross-sectional area of the M. rectus femoris rose by 0.57 cm^2^ in the IT-group. Seymour et al. described the difference of 115 mm^2^ between healthy subjects and COPD patients [22]. In this regard, an increase of 57 mm^2^ (as found in our study) would roughly bisect the difference, which might be considered as relevant. This is further confirmed by earlier data that also demonstrated an increase of cross-sectional area (0.57 cm^2^) of the M. rectus femoris after eight weeks of bilateral high intensity isokinetic knee extensor resistance training [28]. To the best of our knowledge there are no data the correlate the rectus femoris cross sectional area to clinically relevant outcome parameter.

We could not detect any differences in QoL using SGRQ and CAT. This is most likely explained by the low-frequency and the low-intensity nature of both programs. In contrast, higher intensity programs have demonstrated positive effects on health-related quality of life [9,27,29]. It may be speculated that a low intensity training program does not result in effects large enough to measure. In a randomized study of two exercise training programs of different intensity Camillo et al. observed a significant improvement in heart rate variability only after the high-intensity protocol [8]. Effing et al. demonstrated statistically significant between-group differences in exercise capacity and daily activity in an evaluation of the “COPE-active program” [29]. The interventional exercise program consisted of a 6-month “compulsory” period (3 sessions/week) and subsequently a 5-month “optional” period (2 sessions/week). One session/week (control group) consisted of unsupervised home-based exercise training. Of 153 patients, 74 intervention and 68 control patients completed the one-year follow-up. Again, significant effects were seen in a relatively intense program (2 – 3 sessions/week) [29]. Finally, Probst et al. compared the effects of a high-intensity whole-body endurance-and-strength program and a low-intensity calisthenics-and-breathing-exercises program on different outcome parameters [9]. Both groups underwent 3 sessions per week for 12 weeks. Exercise capacity and muscle force significantly improved only in the endurance-and-strength group. Health-related quality of life and functional status improved significantly in both groups. Even the “low-intensity” exercise program included 3 sessions per week [9]. In summary, most “positive studies” published were of higher intensity than the two programs we conducted.

A major problem of all these studies is the inclusion criteria and the compliance of patients. One recent review pointed out that the majority of positive studies did not clarify which patients were included [20]. Only 12% of studies included in this review reported the number of contacted patients. In these studies only 28% completed the program. Altogether 75% of the patients suitable for exercise programs were omitted due to sampling exclusion and dropout. The authors concluded that most of the study populations were not representative of the target population. In general, adherence is a common problem in rehabilitation studies with COPD patients. Drop-out-rates up to 50% are not unusual [5,28-30]. The main causes are often difficult to clarify. By telephone interviews the most often mentioned reasons were disease-related drop-out, disagreement with group assignment, and missing motivation [20]. Missing motivation may be a sign of depression which has been reported to be a frequent comorbidity in COPD [30,31]. No clear recommendation exists how to deal with these frequently occurring problems.

When considering only patients that entered the training period we observed a differential dropout, with significantly more subjects stopping NT than IT (13/27 vs. 5/25; p = 0.04). Assessing possible reasons for this phenomenon, the main causes were not significantly different between both groups (Table 3). To our knowledge, published literature does not provide plausible data to sufficiently explain this issue.

On a closer view it becomes clear that the patients that dropped out had a lower quality of life, worse lung function and elevated inflammatory markers like AAT and CRP (Table 5). In summary, patients with a worse baseline condition had a higher probability to drop out. This is in line with the observation that a higher FEV_1_, CRQ-Score or a shorter distance to the location where training takes place would increase the adherence [28].

We could demonstrate an increase of PGC1-α in the IT group. Since the Irisin values did not show a subsequent increase, the relevance of this result remains unclear. We could not find significant changes in all other measured inflammatory markers and myokines which strengthenes the assumption that low intensity training of 1 hr /week regardless of the modality is not sufficient.

The study has significant limitations. First, we included a relatively small number of patients from a wide range of the disease (GOLD stage I – IV). As a potential training effect might be achievable in some stages of the disease and not in others, this may have influenced the results. On the other hand, as neither the mean FEV_1_ nor the GOLD stages differed significantly between both groups we do not think that the results have biased on a (potentially missed) between-group differences. Secondly, we observed a high dropout rate (as other groups before). This may have attenuated the effect of training. Thirdly, we did not assess depression via standardized questionnaires.

Taken together, this is to our knowledge the first study comparing different low-intensity training approaches in stable COPD patients in a randomized way. The data seem to favor the individualized low frequency training program but do not result in significant improvements of quality of life.

At the moment, it remains unclear how to resolve the discrepancies between guideline recommendations and existing structures. In many countries, we do not have the infrastructure to train our patients 3–5 times per week (as recommended in recent international guidelines [32]). Furthermore a significant proportion of patients would not attend more frequent training opportunities. This results in the need to optimize once weekly training. We believe, that - beside the training approach - the training intensity is an important trigger of success. We conclude, that if low intensity training was chosen and only once weekly training can be proffered, we would suggest to offer an individualized training. The significance of exercise-intensity increase has to be evaluated in further studies.

## Consent

Written informed consent was obtained from the patient for the publication of this report and any accompanying images.

## Competing interests

The project was supported by the German Centre for Lung Research (DZL). The study has been funded partially by GSK. No further conflict of interest has to be acknowledged.

## Authors’ contributions

JK, K Kehr, DH, SF, UK and K Koehler performed experiments, measurements and included patients to the study. ARK, TG, CV, CN and K Kenn contributed to the design, statistics and conception of the study, and contributed to drafting the manuscript. ARK contributed to the design and conception of the study. He included patients, analysed and interpreted the data and drafted the manuscript. All authors read and approved the final manuscript.

## Supplementary Material

Additional file 1 Training Approaches. Click here for file
