# Supplementary material for: A randomized clinical trial to assess the influence of a three months training program (Gym-based individualized vs. Calisthenics-based non-invidualized) in COPD-patients
Source: Respir Res. 2014 Mar 25;15(1):36. doi: 10.1186/1465-9921-15-36 (PMC4021228; doi:10.1186/1465-9921-15-36)
Supplement: Additional file 1 — Training Approaches. [file 1465-9921-15-36-S1.docx]

**Additional file 1**

**Training Approaches**

The training of the two groups (IT and NT) took place once a week for an hour. Location was the department of physiotherapy in the University Hospital of Marburg.

In both cases the group was led by a specially educated physiotherapist. The training was done in both groups by the same physiotherapist. Only during vacation periods and illness the physiotherapist was replaced for both groups. To limit a potential bias in the evaluation, the physiotherapists were not involved in outcome measurement procedures.The duration of the training lasted three to four months, so that the participants could attend at least to 12 trainings sessions. At the beginning and after three months measurements have been performed. All patients were encouraged to perform domestic workout. However, it was not systematically assessed, to which extent this recommendation was followed.

*Non-individualized Training (NT):*

The composition and implementation of the group was based on current recommendations^30^.

Each weekly performed training unit consists out of various parts, which have different aims and do not need to be performed in every unit. One aspect of the NT is the endurance training with different exercises like walking or climbing stairs. Further, the muscle strength was practiced by using dumbbells or thera-bands, based on every day activities. The intensity was patient regulated, meaning that patients repeated the exercises as often they could. Reasons for discontinuation were muscular complaints and/or increasing dyspnoea. Another object of the NT was to improve the coordination capability, the balance as well as the responsiveness of patients. This was implemented with the help of ball games or by using gymnastic balls for short periods of 10 – 15 min. Periodically, the training session included short theory lessons, in which the members gained more knowledge about their disease, the symptoms and the importance of a multimodal therapy concept. Disease-specific techniques (body positions, different breath and cough techniques, like the pursed lips breathing) were communicated to the group.

The structure of one training session was as it follows: The unit always begun with a warm up in the form of free movements in the room for about 10 minutes. Accordingly, at the end of the training units, different relaxing exercises have been accomplished. The mean training consisted of some of the main focuses mentioned above. The decision for a special focus also depended on the typical complaints of the patients in accordance to the seasons. For example, during fall and winter the patients suffer more frequently from exacerbations of the COPD.

*Individualized training (IT)*

The composition of our IT based on the guideline of the ACCP/AACVPR^5^. Each patient received an individual training schedule at the beginning of the training period based on his maximal force and endurance time in different modalities (repetition method of Rühl/Radlinger). The measurement of the endurance capacity was performed on a bicycle ergometer: The patient started to cycle on the ergometer set at 25 watts. After two minutes the exercise load was raised by 5 watts. At the same time, we asked the participants about their subjective estimation regarding the grade of exertion and dyspnea (Borg Scale, ranging from 6 - 20). Simultaneously, the oxygen saturation was monitored. The test was stopped when the patient reached “20” on the Borg Scale or the saturation decreased below 80 %. Based on the results of these first examinations the trainings schedule was made individually for every patient and consisted of different strength and endurance exercises. In the context of the training there was a special focus on the following muscle groups: thigh muscles (especially the quadriceps femoris), lateral hip and trunk stabilizers, anterior shoulder muscles, rotator cuff muscles, different muscles of the upper extremities and dorsal trunk and scapular stabilizers. The intensity of the training changed during the three months of exercise. In the first three to four trainings sessions the participants practiced with 30 % of their maximal muscle strength and about 25 repetitions in three series. After this, the strength training was increased, so that the patients used 40 % to 70 % of their maximal muscle strength with 8 to 15 repetitions in 2 to 6 series.

Endurance training was done on the ergometer as follows: The trainings started with a warm-up, two load phases with duration of three minutes and a cool-down. During the load phases the patients drove at a exercise load, which corresponded to value 13 on the Borg Dyspnoea Scale. Between these more exhausting episodes small breaks were possible, in which the patients cycled at 20 watts.

**Laboratory analyses**

The transcription factor PCG1α was analysed in serum using Western Blot. Each serum sample (diluted 1:300 with phosphate buffered saline) was separated by 10% SDS-Polyacrylamid-gelelectrophoresis, transferred to PVDF membrane and detected with a primary antibody against PCG1-α (polyclonal IgG antibody coupled to HRP (Antibodies-online GmbH) produced in goat, dilution 1:500 in TBST (mixture of Tris-Buffered Saline and Tween 20 ) supplemented with 5% milk powder, incubation over night at 4° C). Detection of enhanced chemiluminescence was performed after treatment with secondary antibody (Anti-goat IgG, peroxidase conjugated (Sigma Aldrich^®^) dilution 1:20000 in TBST with 3% milk powder, 1h at room temperature) with *intas SCIENCE IMAGING* ChemoCam system. Each individual Western blot was directly developed for 10 min. Relative quantification of individual band volumes was performed using *LabImage 1D,* 1D Gel and Western Blot Analysis Software (BIOTEC FISCHER) with normalization to one reference sera per blot.
